# Supplementary material for: Kidney Function Change and All-Cause Mortality in Denosumab Users with and without Chronic Kidney Disease
Source: J Pers Med. 2022 Jan 31;12(2):185. doi: 10.3390/jpm12020185 (PMC8875658; doi:10.3390/jpm12020185)

Table S1. Deyo's Charlson comorbidity index (ICD-9CM and ICD-10-CM)

Comorbid conditions	ICD-9-CM	ICD-10-CM
Myocardial infarction	410.x, 412.x	I21.x, I22.x, I25.2
Congestive heart failure	428.x	I09.9, I11.0, I13.0, I13.2, I25.5, I42.0, I42.5–I42.9, I43.x, I50.x, P29.0
Peripheral vascular disease	443.9, 441.x, 785.4, V43.4	I70.x, I71.x, I73.1, I73.8, I73.9, I77.1, I79.0, I79.2, K55.1, K55.8, K55.9, Z95.8, Z95.9
Cerebrovascular disease	430.x-438.x	G45.x, G46.x, H34.0, I60.x–I69.x
Dementia	290.x	F00.x–F03.x, F05.1, G30.x, G31.1
Chronic pulmonary disease	490.x-505.x, 506.4	I27.8, I27.9, J40.x–J47.x, J60.x–J67.x, J68.4, J70.1, J70.3
Rheumatic disease	710.0, 710.1, 710.4, 714.0-714.2, 714.81, 725.x	M05.x, M06.x, M31.5, M32.x–M34.x, M35.1, M35.3, M36.0
Peptic ulcer disease	531.x-534.x	K25.x–K28.x
Mild liver disease	571.2, 571.4-571.6	B18.x, K70.0–K70.3, K70.9, K71.3–K71.5, K71.7, K73.x, K74.x, K76.0, K76.2–K76.4, K76.8, K76.9, Z94.4
Diabetes without chronic complication	250.0–250.3, 250.7	E10.0, E10.1, E10.6, E10.8, E10.9, E11.0, E11.1, E11.6, E11.8, E11.9, E12.0, E12.1, E12.6, E12.8, E12.9, E13.0, E13.1, E13.6, E13.8, E13.9, E14.0, E14.1, E14.6, E14.8, E14.9

Diabetes with complication	250.4–250.6	E10.2–E10.5, E10.7, E11.2–E11.5, E11.7, E12.2–E12.5, E12.7, E13.2– E13.5, E13.7, E14.2–E14.5, E14.7
Hemiplegia or paraplegia	344.1, 342.x	G04.1, G11.4, G80.1, G80.2, G81.x, G82.x, G83.0–G83.4, G83.9
Renal disease	582.x , 583-583.7 , 585.x , 586.x , 588.x	I12.0, I13.1, N03.2–N03.7, N05.2–N05.7, N18.x, N19.x, N25.0, Z49.0–Z49.2, Z94.0, Z99.2
Any malignancy, including lymphoma and leukemia, except malignant neoplasm of skin	140.x-172.x, 174.x-195.8, 200.x-208.x	C00.x–C26.x, C30.x–C34.x, C37.x– C41.x, C43.x, C45.x–C58.x, C60.x– C76.x, C81.x–C85.x, C88.x, C90.x–C97.x
Moderate or severe liver disease	456.0-456.21, 572.2-572.8	I85.0, I85.9, I86.4, I98.2, K70.4, K71.1, K72.1, K72.9, K76.5, K76.6, K76.7
Metastatic solid tumor	196.x-199.1	C77.x–C80.x
AIDS/HIV	042.x-044.x	B20.x–B22.x, B24.x

Table S2. Definition of a specific disease in this study

Definition	ICD-9-CM	ICD-10-CM
All fractures	800-829	S02,S12,S22,S32,S42,S49,S52,S59, S62,S69,S72,S79,S82,S89,S92,S99
Hip fracture	820.x	S72, S79
Hip surgery	84.17, 79.25, 79.35, 79.05, 79.15, 79.09, 79.19, 03.53, 80.15, 80.75, 80.95, 79.85, 79.75, 83.14, 83.19, 84.19, 79.39, 79.29, 81.51, 81.52, 81.54, 81.81, 81.40, 81.21, 81.53, 77.85, 80.05, 79.29, 79.39, 77.15	Operation procedure code 0SJ, 0MC, 0S9, 0SC, 0Y6, 0QS, 0PS, 0M9, 0SB, 0MB, 0MT, 0ST, 0SS, 0J8, 0JD, 0Q9, 0SR, 0RR, 0SQ, 0SG, 0SW, 0QB, 0QT, 0SP
Hemodialysis	585 plus hemodialysis treatment procedure code (58001C, 58019C, 58020C, 58021C, 58022C, 58023C, 58024C, 58025C, 58026C, 58027C, 58029C) for more than 3 months	N186 plus hemodialysis treatment procedure code (58001C, 58019C, 58020C, 58021C, 58022C, 58023C, 58024C, 58025C, 58026C, 58027C, 58029C) for more than 3 months
Peritoneal dialysis	585 plus hemodialysis treatment procedure code (58002C, 58009B, 58010A, 58010B, 58011A, 58011AB, 58017B, 58017C, 58011B, 58011C, 58012A, 58012B, 58028C) for more than 3 months	N186 plus hemodialysis treatment procedure code (58002C, 58009B, 58010A, 58010B, 58011A, 58011AB, 58017B, 58017C, 58011B, 58011C, 58012A, 58012B, 58028C) for more than 3 months

Table S3. Definition of medications

Medication group	Specific medications	ATC code
Osteoporosis drug	Denosumab	M05BX04
Osteoporosis drug	Etidronic acid, clodronic acid, pamidronic acid, alendronic acid, tiludronic acid, ibandronic acid, risedronic acid, zoledronic acid, strontium ranelate, raloxifene, teriparatide	M05BA, M05BB, M05BX03, M05BX53, G03XC01, H05AA02
Diabetic drugs	Insulin, Biguanides, Sulfonylureas, Alpha-glucosidase inhibitors, Thiazolidinediones, Dipeptidyl peptidase 4 (DPP-4) inhibitors, Glucagon-like peptide-1 (GLP-1) analogues, Sodium-glucose cotransporter-2 (SGLT-2) inhibitor	A10A, A10B
Antihypertensive	Angiotensin-Converting Enzyme inhibitors (ACEI), Angiotensin Receptor Blockers (ARB), Renin-inhibitors, Beta-blockers, Calcium channel blockers, Alpha-blocker	C02CA, C07, C08, C09A, C09B, C09C, C09D, C09XA
Lipid-lowering drugs	HMG CoA reductase inhibitors, Fibrate	C10AA, C10AB
Anticoagulants	Warfarin, Direct factor Xa inhibitors, Direct thrombin inhibitors	B01AA03, B01AE, B01AF
Diuretics	Thiazide, Loop diuretics, Potassium-sparing angents, Vasopression antagonists	C03A, C03C, C03D, C03XA
Proton pump inhibitors	Proton pump inhibitors	A02BC
Nonsteroidal anti-inflammatory drugs (NSAIDs)	Traditional Nonsteroidal anti-inflammatory drugs (NSAIDs), Cox-2 inhibitor	M01AA, M01AB, M01AC, M01AE, M01AG, M01AH
Corticosteroids	Mineralocorticoids, Glucocorticoids	H02AA, H02AB

Table S4. Definition of the cause of death

Cause of death	ICD-10 code
Cardiovascular mortality	I01-I02.0, I05-I09, I20-I25, I27, I30-I52, I60-I69, I70, I71
Non-cardiovascular mortality	C00-C97, D00-D48, D50-D64, E10-E14, F01-F03, G00, G03, G12, G20-G21, G30, I10-I15, J10-J11, J12-J18, J20-J21, J40-J47, J60-J65, J66, J68-J69, K25-K28, K40-K46, K56, K70, K73-K74, K80-K82, L00-L99, M00-M99, N00-N07, N17-N19, N25-N27, O00-O99, P00-P96, Q00-Q99, R54, R95, V01-X59, Y85-Y86, X60-X84, Y87.0, X85-Y09, Y87.1

Table S5. Specific comorbidities in Deyo's Charlson comorbidity index between high and low adherence denosumab users in the overall cohort

	Low adherence (n = 409)	High adherence (n = 713)	*p*-value
Myocardial infarction	15 (3.7%)	26 (3.6%)	1.00
Congestive heart failure	38 (9.3%)	54 (7.6%)	0.37
Peripheral vascular disease	9 (2.2%)	29 (4.1%)	0.14
Cerebrovascular disease	69 (16.9%)	134 (18.8%)	0.47
Dementia	46 (11.2%)	64 (9.0%)	0.26
Chronic pulmonary disease	51 (12.5%)	110 (15.4%)	0.20
Rheumatic disease	14 (3.4%)	29 (4.1%)	0.70
Peptic ulcer disease	86 (21.0%)	161 (22.6%)	0.60
Mild liver disease	33 (8.1%)	66 (9.3%)	0.57
Moderate or severe liver disease	1 (0.2%)	4 (0.6%)	0.66
Diabetes without chronic complication	149 (36.4%)	222 (31.1%)	0.08
Diabetes with complication	60 (14.7%)	88 (12.3%)	0.31
Renal disease	66 (16.1%)	96 (13.5%)	0.26
Hemiplegia or paraplegia	6 (1.5%)	10 (1.4%)	1.00
Any malignancy	55 (13.4%)	91 (12.8%)	0.81
Metastatic solid tumor	5 (1.2%)	9 (1.3%)	1.00
AIDS/HIV	0	0	1.00

Abbreviation: AIDS/HIV, acquired immunodeficiency syndrome/human immunodeficiency virus

Table S6. The crude and adjusted hazard ratio of cardiovascular mortality and non-cardiovascular mortality between high adherence and adherence denosumab treatment in the overall cohort and chronic kidney disease subcohort

	Mortality		Hazard ratio (95% Confidence Interval)	
	No	Yes		
	N(%)	N(%)	Crude	Adjusted*
Cardiovascular mortality				
Overall cohort				
Low adherence (n = 409)	392 (95.8%)	17 (4.2%)	Reference	Reference
High adherence (n = 713)	695 (97.5%)	18 (2.5%)	0.58 (0.30, 1.12)	0.58 (0.30, 1.12)
CKD subcohort				
Low adherence (n = 201)	186 (92.5%)	15 (7.5%)	Reference	Reference
High adherence (n = 299)	287 (96.0%)	12 (4.0%)	0.49 (0.23, 1.04)	0.46 (0.21, 0.98)
Non-Cardiovascular mortality				
Overall cohort				
Low adherence (n = 409)	342 (83.6%)	67 (16.4%)	Reference	Reference
High adherence (n = 713)	633 (88.8%)	80 (11.2%)	0.65 (0.47, 0.90)	0.66 (0.47, 0.91)
CKD subcohort				
Low adherence (n = 201)	155 (77.1%)	46 (22.9%)	Reference	Reference
High adherence (n = 299)	247 (82.6%)	52 (17.4%)	0.69 (0.46, 1.03)	0.66 (0.44, 0.98)

*Adjusted for age, gender, and Charlson comorbidity index score

Table S7. A comparison of ionized calcium level before and after denosumab treatment in the overall cohort and chronic kidney disease cohort

	Before index date	After index date	*p* for paired t-test
Overall cohort			
Low adherence	4.67 ± 0.37 mg/dl	4.38 ± 0.40 mg/dl	0.08
High adherence	4.68 ± 0.29 mg/dl	4.59 ± 0.28 mg/dl	0.10
CKD subcohort			
Low adherence	4.66 ± 0.38 mg/dl	4.33 ± 0.39 mg/dl	0.17
High adherence	4.66 ± 0.32 mg/dl	4.60 ± 0.33 mg/dl	0.29

Ionized calcium level: three months average before the first denosumab treatment (before the index date) and three months average after the first denosumab treatment (after the index date)

Abbreviation: CKD, chronic kidney disease

Figure S1. Kaplan Meier curve for cardiovascular mortality, cancer mortality, infectious mortality, and other mortality between high adherence and low adherence of denosumab treatment in (A) overall cohort and (B) chronic kidney disease subcohort

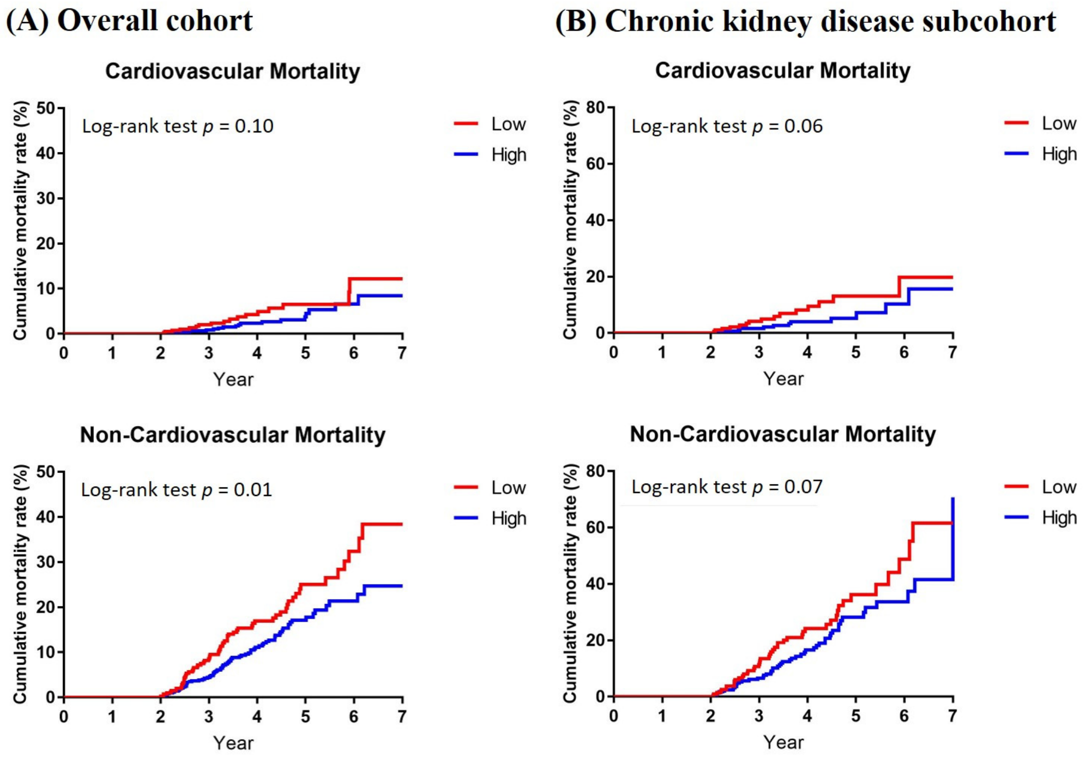

Supplement: Supplementary file 1 [file jpm-12-00185-s001.zip › jpm-1530381-supplementary.pdf]
